# Functional Subdivisions of the Cerebellum in Naturalistic Paradigm Functional Magnetic Resonance Imaging

**DOI:** 10.3389/fnins.2021.748561

**Published:** 2021-12-17

**Authors:** Jianing Hao, Xintao Hu, Liting Wang, Lei Guo, Junwei Han

**Affiliations:** School of Automation, Northwestern Polytechnical University, Xi’an, China

**Keywords:** cerebellum, subdivision, functional magnetic resonance imaging, naturalistic paradigm, data driven, cerebellum–cerebrum connectivity

## Abstract

Compelling evidence has suggested that the human cerebellum is engaged in a wide range of cognitive tasks besides traditional opinions of motor control, and it is organized into a set of distinct functional subregions. The existing model-driven cerebellum parcellations through resting-state functional MRI (rsfMRI) and task-fMRI are relatively coarse, introducing challenges in resolving the functions of the cerebellum especially when the brain is exposed to naturalistic environments. The current study took the advantages of the naturalistic paradigm (i.e., movie viewing) fMRI (nfMRI) to derive fine parcellations *via* a data-driven dual-regression-like sparse representation framework. The parcellations were quantitatively evaluated by functional homogeneity, and global and local boundary confidence. In addition, the differences of cerebellum–cerebrum functional connectivities between rsfMRI and nfMRI for some exemplar parcellations were compared to provide qualitatively functional validations. Our experimental results demonstrated that the proposed study successfully identified distinct subregions of the cerebellum. This fine parcellation may serve as a complementary solution to existing cerebellum parcellations, providing an alternative template for exploring neural activities of the cerebellum in naturalistic environments.

## Introduction

Compelling evidence from anatomy, clinical, behavioral, and neuroimaging studies indicates that the human cerebellum is engaged in cognitive and affective processing beside traditional opinions of motor control (Ivry and Baldo, 1992; Strick et al., 2009; Buckner, 2013; Klein et al., 2016; Schmahmann et al., 2019). Subdividing of the cerebellum into a discrete set of regions and comprehensively mapping of their functions have gained increasing interests in recent years (O’Reilly et al., 2010; Buckner et al., 2011; King et al., 2019; Xue et al., 2020).

The homogeneity of cytoarchitectonic organization and the difficulty of tracing cortico-cerebellar anatomical connections in the cerebellum bring considerable limitations in cerebellum parcellation *via* neuroimaging and neuropsychological approaches (O’Reilly et al., 2010; Buckner et al., 2011; King et al., 2019). Thus, several strategies based on functional MRI (fMRI) have been proposed. Using resting-state fMRI (rsfMRI), seed-region-based cerebellum–cerebrum functional connectivity studies revealed two systems in the cerebellum included an inverted somatomotor map in the anterior lobe and a second inversion posterior map between the anterior and posterior representations (O’Reilly et al., 2010). According to the cerebral functional brain networks discovered *via* rsfMRI, more detailed parcellations (e.g., 17 regions) have been achieved based on voxel-wise cerebellum–cerebrum functional connectivity analysis. These studies have suggested a third system in the cerebellum that is associated with homotopic maps of the full cerebrum (Buckner et al., 2011), and a separate representation of a language network (Xue et al., 2020). The subdivisions and their functional interpretations in rsfMRI-based studies highly rely on functional network mapping of the cerebrum. In addition to rsfMRI, the functional subdivisions in the cerebellum have been assessed using massive task fMRI (tfMRI) that included a battery of 26 diverse tasks comprising 47 unique conditions (King et al., 2019). TfMRI-based parcellations have the advantage over the rsfMRI ones in that task conditions can be used as benchmarks to characterize the cognitive processes related to the subdivisions. However, it is largely unknown how the cerebellum functions when the brain is exposed to complex perceptual environments where multiple task-demands coexist and functional interactions among multiple brain systems are essential.

In recent years, naturalistic paradigm (e.g., movie viewing or music listening) fMRI (nfMRI) has emerged as an ecologically valid tool to map brain functions that approximate real-life experience (Hasson et al., 2004; Golland et al., 2007; Hasson and Honey, 2012; Hu et al., 2016). Compared with abstract task paradigms and resting-state, naturalistic paradigms can not only enhance reliable neural activities in a wide range of functionally specialized areas (Hasson et al., 2010) but also engage a broader set of functional interactions between multiple brain systems (Bartels and Zeki, 2005; Wang et al., 2016). More importantly, the activations in some brain regions (e.g., the default mode network (DMN) as a spatial case) are in favor of such dynamic stimuli (Yeshurun et al., 2021). In addition, naturalistic paradigms are equipped with rich, dynamic, and multimodal stimuli (e.g., visual, auditory/language, and emotion). Thus, it is more feasible to relate neural activities to cognitive processes and behavioral measurements compared with rsfMRI. Thus, nfMRI may serve as a feasible tool to identify the functional boundaries of the cerebellum complementary to rsfMRI and tfMRI.

In this study, functional subdivisions of the cerebellum were parcellated *via* a dual-regression-like framework based on sparse representation of nfMRI from the Human Connectome Project (HCP). The parcellations were quantitatively evaluated and validated by functional homogeneity and boundary confidence, and quantitatively by comparison between cerebellum–cerebrum functional connectivity patterns in nfMRI and rsfMRI. Our experimental results demonstrated that 64 distinct subregions of the cerebellum can be successfully identified, and the functional homogeneity and boundary confidence can be largely improved compared with existing parcellations.

## Materials and Methods

### Data Set and Preprocessing

The HCP has released two phases of 7T movie-watching fMRI datasets (Van Essen et al., 2012). The movie stimuli are short independent film and Hollywood movie excerpts concatenated into four video clips. All the four video clips include a Vimeo repeat clip to be used for validation across scans. The nfMRI data used in the present study are corresponding to the repeat clip in the first scan of the second phase. The released movie-watching data were preprocessed using the HCP minimum preprocessing pipelines (Glasser et al., 2013). The cerebellum voxels (8,709 voxels in the left and 9,144 voxels in the right cerebellum) were defined according to the gray ordinate spatial coordinate system. The nfMRI data were further spatially smoothed using a 4-mm full-width-half-maximum Gaussian kernel. NfMRI sequences of 60 randomly selected subjects form the developing dataset (Dataset-D) and those of 20 randomly selected subjects form the validation dataset (Dataset-V).

### Cerebellum Parcellation *via* Dual-Regression-Like Sparse Representation

Dual regression is a widely used framework for data-driven fMRI analysis (Svensen et al., 2002; Egolf et al., 2004; Du and Fan, 2013). It first runs a group-wise fMRI data decomposition to get a set of common spatial maps shared by all subjects. Then for each subject, these spatial maps are used as templates in a spatial regression stage to identify subject-specific time courses. The subject-specific time courses are fed to a temporal regression stage to identify subject-specific spatial maps.

In this study, group-wise spatial maps were derived *via* a well-established data-driven fMRI blind component separation approach based on sparse representation (Lv et al., 2015). More specifically, dictionary learning and sparse representation algorithm (Mairal et al., 2009) was applied to the temporally concatenated fMRI sequences of multiple subjects in Dataset-D. This returned a dictionary in which each atom characterizes a representative temporal activity pattern embedded in the input fMRI data and a set of spatial maps depicting the spatial distribution of corresponding temporal activities. Subsequently, these spatial maps were used as templates in dual regression to estimate subject-specific time courses and spatial maps. A one-sample *t*-test followed by Bonferroni false discovery rate (FDR) correction was performed on the aggregated subject-specific spatial maps to get a *z*-scored significance map for each fMRI component. Afterward, a global threshold was applied to all the significance maps to achieve the parcellations. Structural symmetry is a common qualitative metric in fMRI analysis especially when the ground truth is known. Thus, the cerebellum parcellation framework described earlier was applied to the left and right cerebellum independently.

The parameter of dictionary size in the dual-regression-like framework based on sparse representation algorithm defines the expected number of components to separate. In this study, it was empirically set as 32 through intensive experiments. That is, 32 regions were parcellated in both the left and right cerebellum.

### Evaluation and Comparison Studies

Several experiments were conducted to evaluate the parcellations quantitatively using Dataset-V. First, the functional homogeneity of those parcellations were measured *via* principal component analysis (PCA). For each subject, PCA was applied on the nfMRI time courses of each parcellation, resulting in a set of orthogonal eigenvectors representing principal components embedded in the time courses. The ratio between the first eigenvalue and the summation of all the eigenvalues indicates the percentage of variation that can be explained by the first principal component, reflecting functional homogeneity of the parcellation. In addition, the confidence of parcellation boundaries was measured using distance-controlled boundary coefficient (DCBC; King et al., 2019). The global DCBC measures the difference between correlations of all possible within-region and between-region voxel pairs using a range of spatial bins (3 to 30 mm with interval of 3 mm). The advantage of DCBC is extending standard clustering metrics to account for spatial distance. A positive DCBC indicates that voxel pairs located in the same region are more functionally related than voxel pairs that lie across boundaries. The local DCBC was used to evaluate the confidence of individual boundaries. A local DCBC was calculated only on the voxel pairs from the regions that are separated by that boundary.

The dual-regression-like sparse representation approach described earlier was applied to resting-state fMRI data to derive a set of parcellations (denoted as Resting-state 64). Functional homogeneity, and the global and local DCBC of our parcellations (denoted as Movie 64) were compared with those of the 17 parcellations based on cerebellum–cerebrum resting-state functional connectivity (denoted as Resting-state 17) (Buckner et al., 2011), 10 parcellations based on multiple domain task battery (MDTB) (denoted as MDTB 10) (King et al., 2019), and Resting-state 64.

### Comparison Between Cerebellum–Cerebrum Connectivities in nfMRI and Resting-State fMRI

The comparison between cerebellum–cerebrum connectivities in nfMRI and rsfMRI on the one hand may serve as evidence to validate the parcellations and on the other hand may provide heuristics to infer the functional processes associated with the subdivisions. For each subject in Dataset-V, a spatial regression strategy was used to extract subject-specific time courses. For each subdivision, the group-wise spatial map in sparse representation analysis was masked by the corresponding binary cerebellum parcellation and were used as input in the spatial regression of the subject-specific nfMRI sequence, resulting in a subject-specific time course for the current subject. The Pearson correlation coefficients between this time course and the time courses of vertices on the cortical surface were calculated to measure the parcellation-specific cerebellum–cerebrum functional connectivities. This procedure was repeated for both nfMRI and rsfMRI of each subject. A two-tailed two-sample *t*-test followed by Bonferroni FDR correction was conducted to infer the difference between the cerebellum–cerebrum functional connectivity patterns of nfMRI and rsfMRI.

## Results

### Functional Subdivisions of the Cerebellum in nfMRI

**FIGURE 1:**
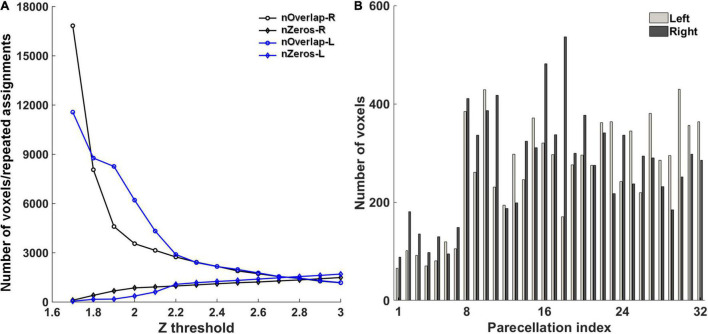
**(A)** The optimization of the global *z*-score threshold in the parcellation. nOverlap-L/R: the number of repeated assignments in the left/right cerebellum. nZeros-L/R: the number of voxels that were not assigned to any parcellation in the left/right. **(B)** The number of voxels in each parcellation.

The first step toward cerebellum parcellation was to determine the global threshold for *z*-scored significance maps. A small threshold brings spatial overlaps between fMRI components, that is, a single voxel may be assigned to multiple components. On the contrary, some voxels would not be assigned to any component if a large threshold is used. In this study, the threshold was optimized to balance the number of repeated assignments among all the components and the number of unassigned voxels, preferring decreasing the number of unassigned voxels. In the experiments, the global threshold was varied from 1.6 to 3 with an interval of 0.1, and the number of repeated assignments and the number of unassigned voxels were counted in each condition (Figure 1A). Accordingly, the global threshold of 2.0 and 1.8 were used for the left and right cerebellum, respectively. With this setting, the number of unassigned voxels was 364 and 407 for the left and right cerebellum, respectively, while the number of repeated assignments was 8,060 and 3,555 for the left and right cerebellum, respectively. The number of voxels in each parcellation with the optimal global thresholds is depicted in Figure 1B.

**FIGURE 2:**
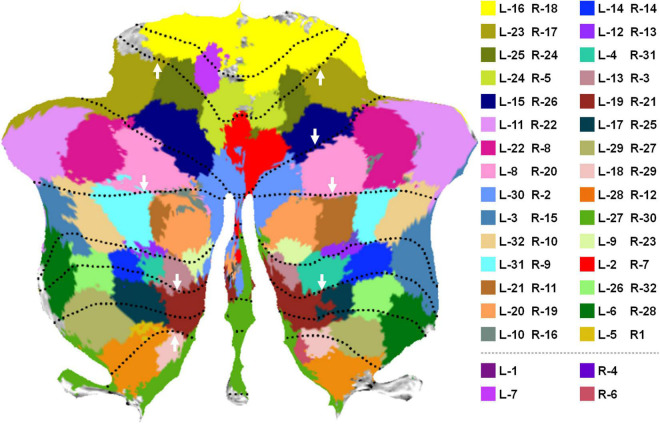
Parcellations were visualized on a flattened cerebellum map. Parcellation boundaries that well matched the lobular boundaries were highlighted by white arrows.

In all the 64 parcellations, we manually selected 30 pairs of parcellations that were spatially symmetric in the left and right cerebellum, as visualized by a flattened cerebellum map in Figure 2 using the SUIT toolbox (Diedrichsen et al., 2009; Diedrichsen and Zotow, 2015). Each pair of regions was color-coded using the same color. Part of the regions without color-coding was due to voxel missing in the released HCP data and the voxels that were not assigned to any parcellation in the thresholding of significance maps (see Supplementary Figure 1 for details). It is notable that the flap map is not a true unfolding of the cerebellar cortex, but retains a roughly proportional relationship between the surface area of the 2D representation and the volume of the underlying cerebellar gray matter. Thus, each pixel on the flat map may correspond to multiple voxels in the volumetric space, resulting in repeated parcellation labels during flap map visualization. In this study, the label of each pixel in the flat map was determined using the most-often occurring value. The volumetric visualization of the parcellations (Supplementary Figure 2) and the flat maps of individual parcellations (Supplementary Figure 3) were also provided as additional visualization. The symmetry of the parcellations on the left and right cerebellum was relatively good by visual inspection. It is also seen that some parcellations well match the major cerebellum lobular boundaries as highlighted by white arrows in Figure 2. Some parcellations span two or more lobules, as cerebellar lobules do not well reflect functional subdivisions (King et al., 2019). These observations provide qualitative evaluation and validation of the fine parcellations proposed in this study.

### Functional Homogeneity and Boundary Confidence

**FIGURE 3:**
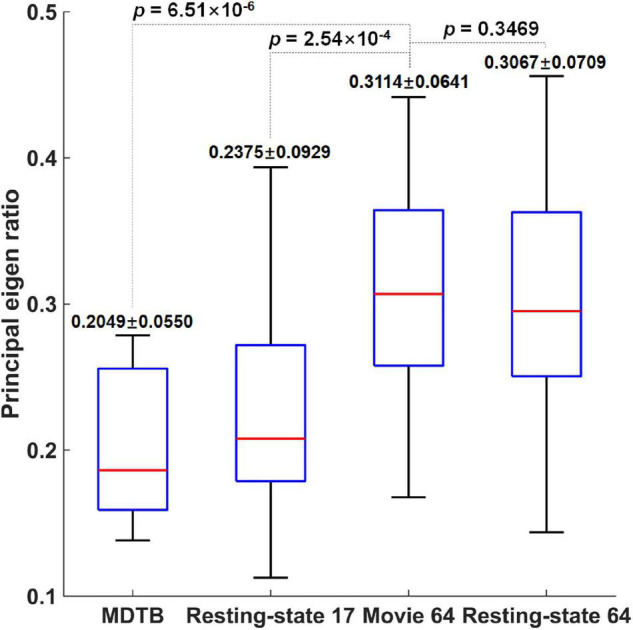
The comparison of functional homogeneity measured by the principal eigen ratio.

Not surprisingly, the Movie 64 parcellation in this study significantly increased average functional homogeneity compared with the MDTB 10 parcellation (*p* = 6.51 × 10^–6^) and Resting-state 17 parcellation (*p* = 2.54 × 10^–4^) as shown in Figure 3. The functional homogeneity in Movie 64 was slightly but not significantly (*p* = 0.3469) higher than that of Resting-state 64. These observations highlighted the necessity of refined parcellations of the cerebellum.

**FIGURE 4:**
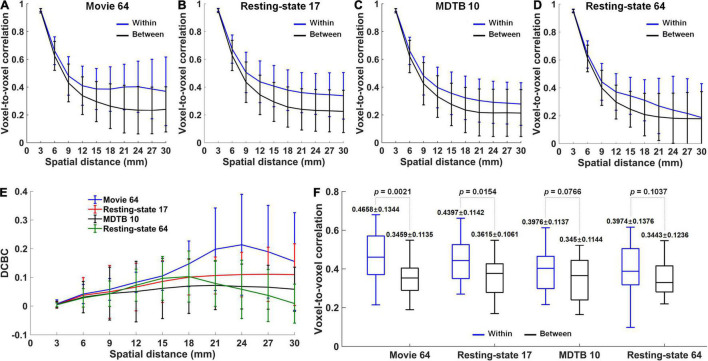
**(A–D)** The correlations averaged over participants for within-region (blue) and between-region (black) voxel pairs in Movie 64, Resting-state 17, MDTB 10 and Resting-state 64 parcellations, respectively. **(E)** The DCBC values in the four parcellations. **(F)** The differences between within-region and between-region correlations averaged over spatial distance bins in the four parcellations.

When averaging over participants, the correlations of within-region voxel pairs were much greater than the correlations of between-region voxel pairs for Movie 64 parcellations, as well as Resting-state 17, MDTB 10, and Resting-state parcellations (Figures 4A–D). Notably, the DCBC values were the largest in Movie 64 parcellation in all the spatial distance bins (Figure 4E). Meanwhile, the Movie 64 parcellation was with the highest within-region correlation, and comparable between-region correlation compared with Resting-state 17, MDTB 10, and Resting-state 64 when averaging over spatial bins (Figure 4F). Two-sample *t*-tests revealed the most significant difference between within- and between-region correlations in Movie 64 parcellations (*p*-value, 0.0021 *vs.* 0.0154 *vs.* 0.0766 *vs.* 0.1037). These observations demonstrated the superiority of cerebellum parcellation presented in the current study.

**FIGURE 5:**
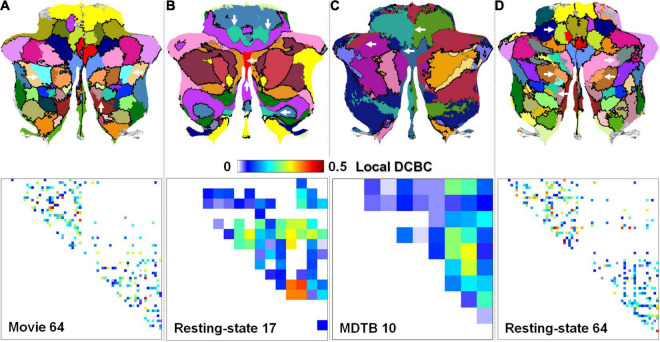
The visualization of the local DCBCs of individual boundaries on flat maps (upper row) and using DCBC matrices (lower row). **(A–D)** are for the Movie 64, Resting-state 17, MDTB 10, and Resting-state 64 parcellations, respectively. White arrows highlight the negative local DCBC values.

**FIGURE 6:**
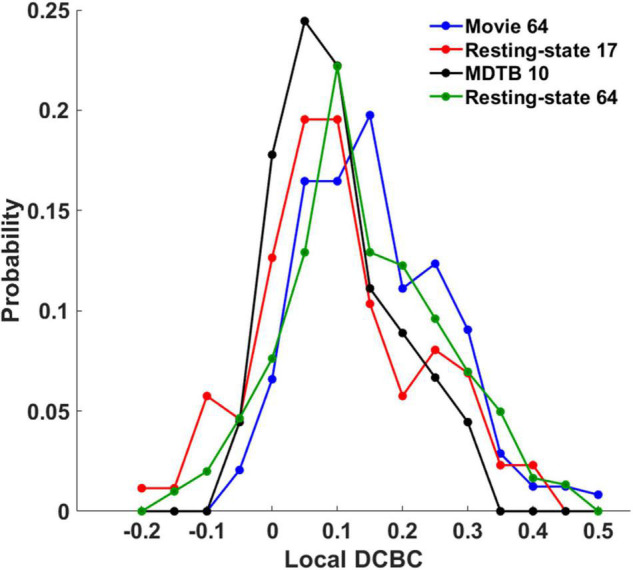
The histograms of the local DCBC values in the four parcellations.

The local DCBC was used to evaluate the confidence of individual boundaries. Figures 5A–D show the qualitative visualization of individual boundaries on the flat map in which the thickness of the boundaries was based on its local DCBC value (the upper row) and the quantitative visualization of the individual boundaries using a DCBC matrix (the lower row) for the four parcellations, respectively. The maximum and minimum in the DCBC matrices are −0.049/0.506, −0.2008/0.4056, −0.0427/0.3033, and −0.17/0.456 for the four parcellations, respectively. It is notable that negative local DCBCs were removed in the DCBC matrices for better visualization. Those negative DCBCs were highlighted by white arrows in the flat maps where boundaries were missing. The proportions of negative local DCBCs were 0.0576 (14 out of in total 243 boundaries), 0.195 (17/87), 0.044 (2/45), and 0.1159 (35/302) in the Movie 64, Resting-state 17, MDTB 10, and Resting-state 64 parcellations, respectively. Detailed histograms (Figure 6) showed that the Movie 64 parcellation was with a larger proportion of higher local DCBC values as its histogram shifted toward the right *x*-axis compared with the remaining three parcellations. These results provided additional evidence of the superiority of the Movie 64 parcellation.

### Comparison Between Cerebellum–Cerebrum Connectivity in nfMRI and Resting-State fMRI

**FIGURE 7:**
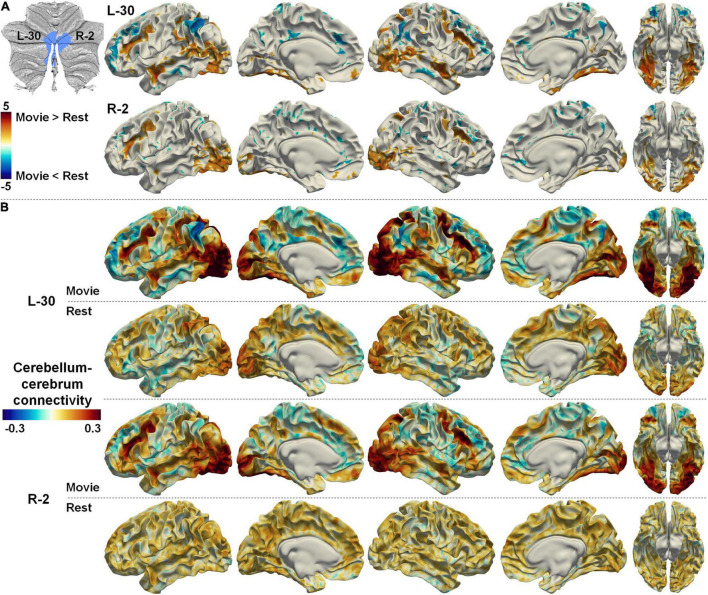
**(A)** The difference between cerebellum–cerebrum connectivities in nfMRI and rsfMRI for two symmetrical regions (L-30 and R-2, related to visual) in the Movie 64 parcellation. **(B)** The strength of cerebellum–cerebrum connectivities for the two regions in nfMRI and rsfMRI.

Full description of the difference between cerebellum–cerebrum connectivities in nfMRI and rsfMRI for all the parcellations is out of the scope of the current study. We only illustrated a few examples. Two symmetrical regions (#30 on the left cerebellum and #2 on the right cerebellum, denoted as L-30 and R-2 hereafter) in the Movie 64 parcellations are thought to be associated with visual processing according to MDTB 10 parcellations. In accordance, the functional connectivities between L-30/R-2 and the bilateral visual cortices (both dorsal and ventral) as well as the dorsal spatial attention cortices (bilateral intra-parietal sulcus and frontal eye fields, IPS and FEFs) were significantly increased while their connectivities to the DMN were significantly decreased in nfMRI compared with those in rsfMRI (Figure 7A). Detailed connectivity strength depicted in Figure 7B shows that the connectivities between L-30/R-2 and visual related cortices were positive in nfMRI but negative in rsfMRI. Meanwhile, the connectivities between L-30/R-2 and the DMN were negative in nfMRI but positive in rsfMRI. These observations may indicate that the functional interactions between L-30/R-2 and DMN were inhibited in nfMRI compared with those in rsfMRI, while the functional interactions between L-30/R-2 and visual related cerebral cortices were promoted in nfMRI compared with those in rsfMRI.

**FIGURE 8:**
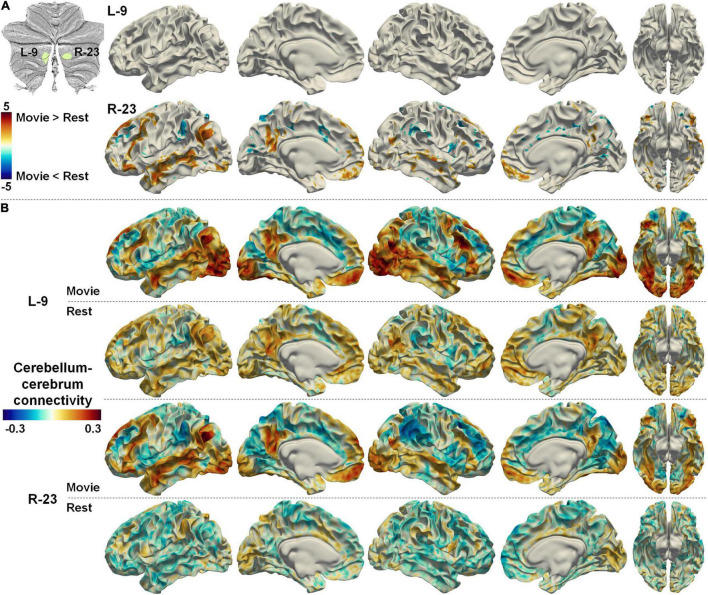
**(A)** The difference between cerebellum–cerebrum connectivities in nfMRI and rsfMRI for two symmetrical regions (L-9 and R-23, related to auditory/language) in the Movie 64 parcellation. **(B)** The strength of cerebellum–cerebrum connectivities for the two regions in nfMRI and rsfMRI.

Two symmetrical regions (L-9 and R-23) in the Movie 64 parcellation were thought to be associated with auditory/language processing according to MDTB 10 parcellation. However, a recent study using individuals with intensively repeated fMRI scans has revealed the right lateralized representation of a language network in the cerebellum (Xue et al., 2020). In agreement with this finding, the functional connectivities between region R-23 and the cerebral language network were significantly increased in nfMRI compared with rsfMRI, while there was no significant difference between connectivities in nfMRI and rsfMRI for L-9 (Figure 8A). The functional connectivities between R-23 and the cerebral language network were positive in nfMRI but negative in rsfMRI, while the functional connectivities between L-9 and the cerebral language network were both positive in rsfMRI and nfMRI (Figure 8B). The cerebellum–cerebrum functional connectivity patterns reported in Figure 7 and Figure 8 provided strong evidence to the reasonability of the Movie 64 parcellation.

**FIGURE 9:**
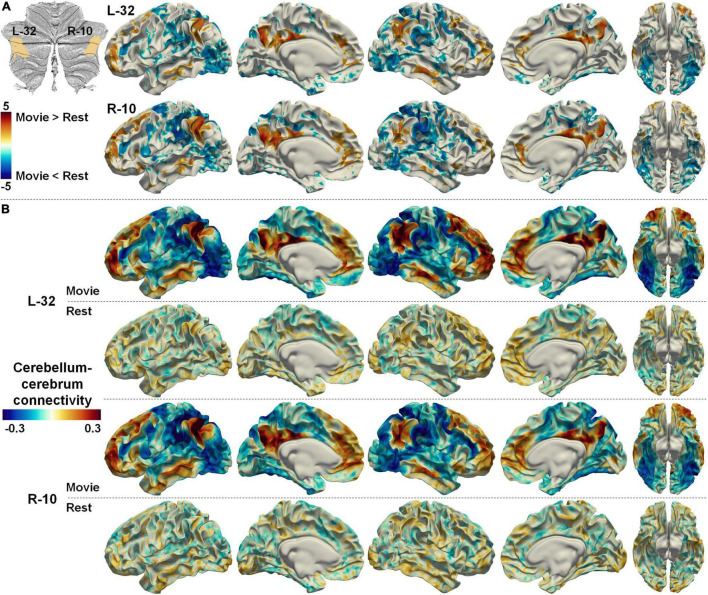
**(A)** The difference between cerebellum–cerebrum connectivities in nfMRI and rsfMRI for two symmetrical regions (L-32 and R-10, related to the DMN) in the Movie 64 parcellations. **(B)** The strength of cerebellum–cerebrum connectivities for the two regions in nfMRI and rsfMRI.

The cerebellum–cerebrum functional connectivity patterns may also provide heuristics to infer the functional processes associated with those parcellations. Two symmetrical regions (L-32 and R-10), which were considered the representation of DMN in the cerebellum (Buckner et al., 2011), were taken as an example. The DMN is classically considered an intrinsic system, decreasing its neural activity in complex attention-demanding tasks (Raichle et al., 2001; Buckner and DiNicola, 2019). However, nfMRI studies has suggested that the DMN plays a central role in integrating incoming extrinsic information with prior intrinsic information over long timescales to form context-dependent models of situations (see a detailed review in Yeshurun et al., 2021). Intriguingly, the functional connectivities between L-32/R-10 and the DMN were significantly increased, while those between L-32/R-10 and unimodal cortices (e.g., visual) were significantly decreased in nfMRI compared with rsfMRI (Figure 9A). Moreover, the spatial patterns of the cerebellum–cerebrum connectivities of those two regions (Figure 9B) largely analog the principal connectivity gradient of the cerebral cortex (Margulies et al., 2016). These observations may indicate the active role that the cerebellum regions play when the brain is exposed to dynamic environments.

## Discussion and Conclusion

The current study proposed a data-driven parcellation of the cerebellum using the naturalistic paradigm fMRI (nfMRI). The parcellations were evaluated and validated via quantitative metrics that included functional homogeneity and boundary confidence. The possible functional processes related to the parcellations were partly accessed by comparison between cerebellum–cerebrum functional connectivities in nfMRI and rsfMRI. Our experimental results demonstrated that the proposed study successfully identified distinct functional subregions of the cerebellum.

The advantages of nfMRI compared with rsfMRI as discussed previously make nfMRI an appropriate tool to map the subdivision of the cerebellum. However, the high functional heterogeneity of the cerebellum (Bloedel, 1992; Stoodley and Schmahmann, 2009) and the complex neural activities evoked by dynamic naturalistic paradigm call for more finer parcellations. The current study adopted a data-driven strategy to address this problem. It is notable that more parcels with smaller region size inherently increase functional homogeneity. Thus, the number of parcels is a confounding factor in statistical comparisons. Nevertheless, the direct comparison of functional homogeneity, and global and local DCBC to Resting-state 64 parcellation showed the superiority of Movie 64 parcellation. The resulted parcellations could be a complementary solution to existing ones, serving as an alternative template for exploring neural activities of the cerebellum in naturalistic environments. In addition, combined with the cortical landmark systems DICCCOL (Dense Individualized and Common Connectivity-based Cortical Landmarks) (Zhu et al., 2013) and A-DICCCOL (Anatomy-guided DICCCOL) (Jiang et al., 2015), the Movie 64 cerebellum parcellation may provide over 1,000 regions to enable large-scale functional brain network analysis to explore how the brain functions in naturalistic environments as a complex networked system.

One limitation of the current study is the lack of functional labels of the parcellations. It is practical to label the parcellations according to the Resting-state 17 or MDTB 10 parcellations depending on spatial overlap criteria. However, those parcellations are relatively coarse. Thus, transferring labels in this way may degenerate the functional specificity of much finer Movie 64 parcellations. The cerebellum–cerebrum connectivity analysis in the current study may provide heuristics to infer underlying functional labels. However, a more comprehensive study is desired in the future to achieve this goal.

**FIGURE 10:**
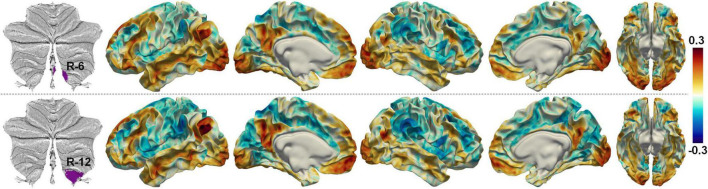
The cerebellum–cerebrum functional connectivities of two regions on the right cerebellum.

Another limitation of the current study is the manual setting of the number of expected parcellations, which is quite common in data-driven methods for blind component identification in fMRI. Although the number of parcellations was carefully set via intensive experiments, the current parcellations may face the risk of over-segmentation as indicated by the negative local DCBCs (Figure 5A). The lowest negative local DCBC value (−0.049) happened at the boundary between parcellations R-6 and R-12 on the right cerebellum. The temporal profiles of these two regions were moderately correlated (*r* = 0.2772, averaged over Dataset-D). Meanwhile, their cerebellum–cerebrum functional connectivity patterns were similar (Figure 10). These observations may suggest that those two regions can be merged as a single one. The over-segmentation problem can be alleviated by an automatic inference of the number of components (e.g., Egolf et al., 2004) in future studies.

## Data Availability Statement

Publicly available datasets were analyzed in this study. This data can be found here: https://db.humanconnectome.org/app/action/DownloadPackagesAction.

## Author Contributions

XH designed the study, analyzed the data, wrote the article, and prepared the figures. JiH analyzed the data and prepared the figures. LW analyzed the data. LG and JuH designed the study and revised the article. All authors contributed to the article and approved the submitted version.

## Conflict of Interest

The authors declare that the research was conducted in the absence of any commercial or financial relationships that could be construed as a potential conflict of interest.

## Publisher’s Note

All claims expressed in this article are solely those of the authors and do not necessarily represent those of their affiliated organizations, or those of the publisher, the editors and the reviewers. Any product that may be evaluated in this article, or claim that may be made by its manufacturer, is not guaranteed or endorsed by the publisher.
