# Supplementary material for: Functional Subdivisions of the Cerebellum in Naturalistic Paradigm Functional Magnetic Resonance Imaging
Source: Front Neurosci. 2021 Dec 17;15:748561. doi: 10.3389/fnins.2021.748561 (PMC8719453; doi:10.3389/fnins.2021.748561)
Supplement: Supplementary file 1 [file Data_Sheet_1.docx]

Supplementary Material

## Supplementary Figures


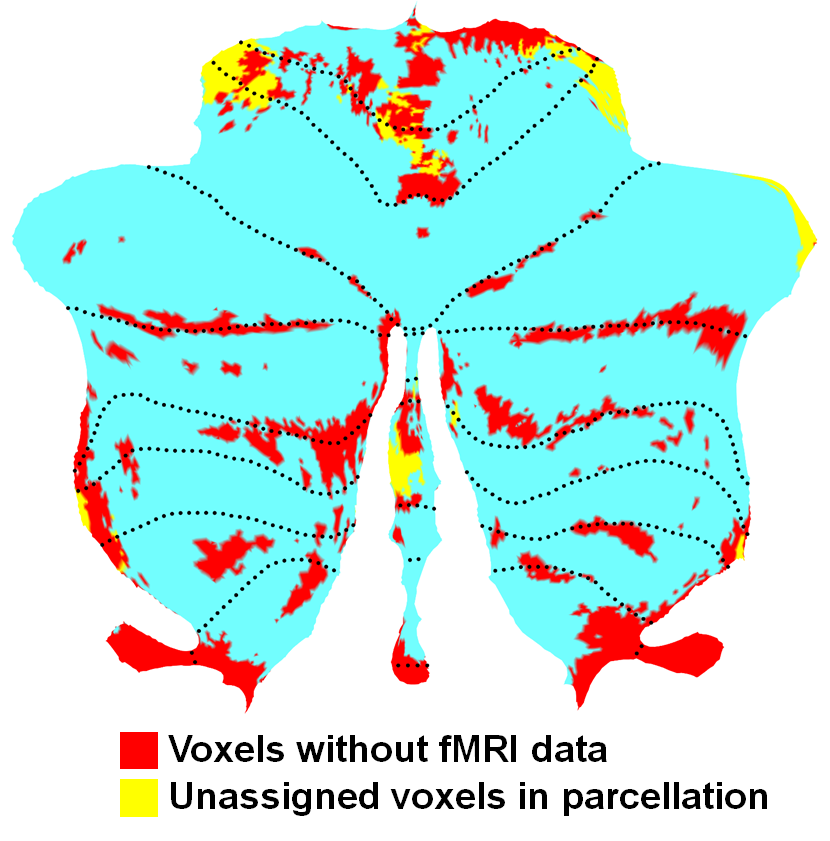


**Supplementary Figure 1.** The distributions of the voxels without fMRI data (red) and the voxels that were not assigned any labels in the parcellations (yellow).


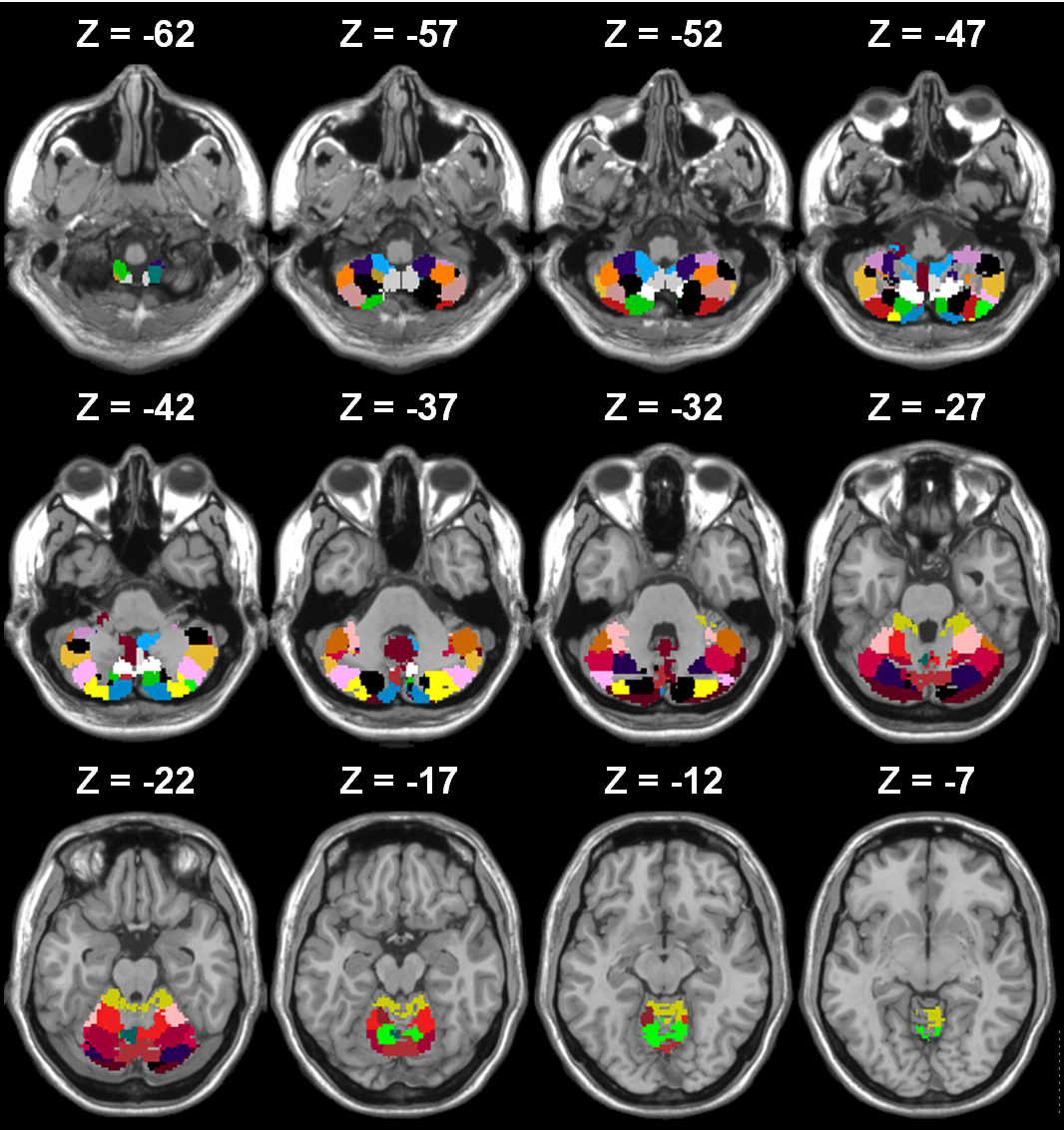


**Supplementary Figure 2.** The volumetric visualization of the parcellations.


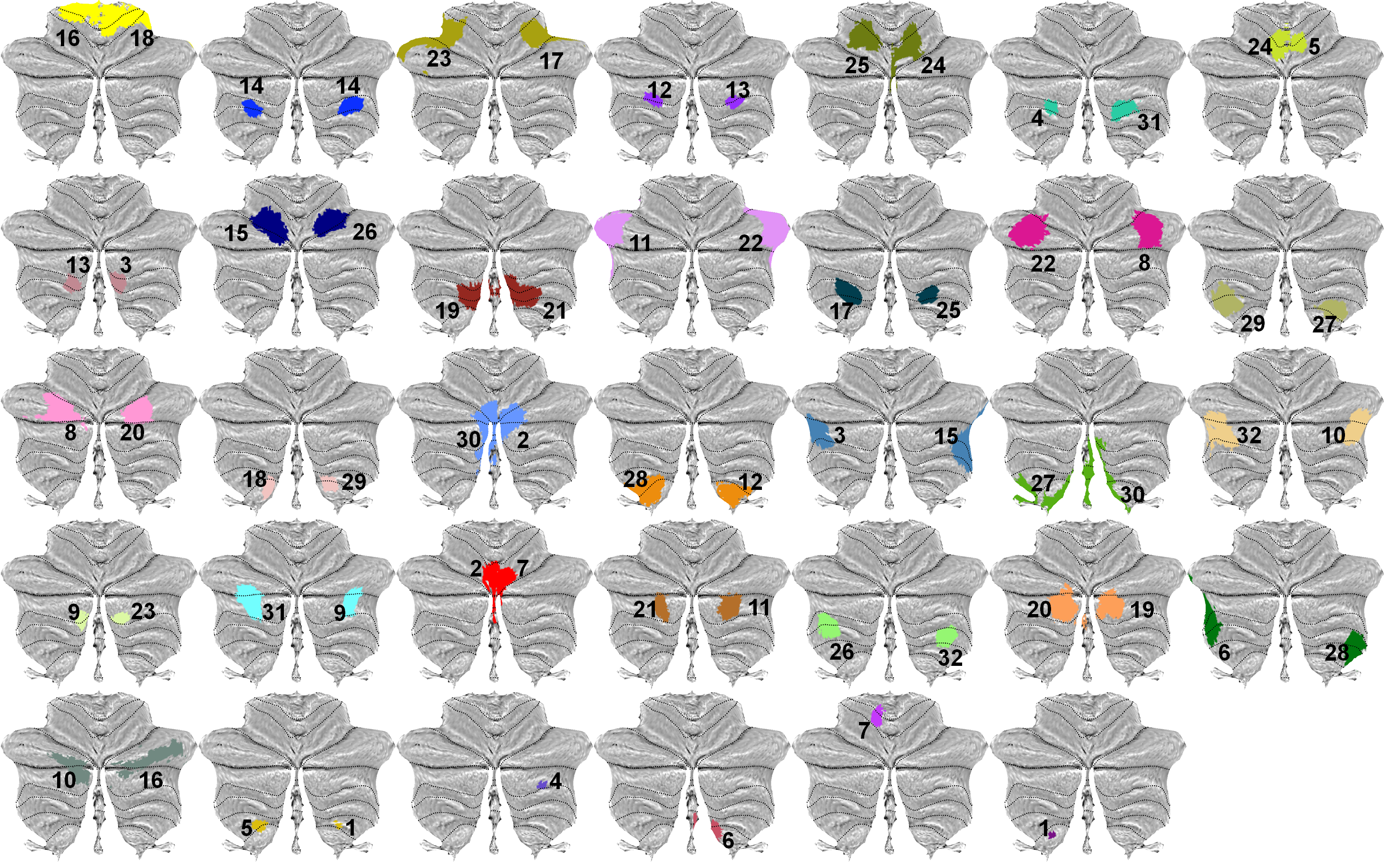


**Supplementary Figure 3.** The flap map visualization of individual parcellation. The pair of regions that are spatially symmetric were color-coded using the same color. Four parcellations were lack of symmetry on the contralateral side (row 5, column 3-6).
